# Self-activated *in vivo* therapeutic cascade of erythrocyte membrane-cloaked iron-mineralized enzymes

**DOI:** 10.7150/thno.39621

**Published:** 2020-01-12

**Authors:** Wen Liu, Miao-Liang Ruan, Lamei Liu, Xin Ji, Yandong Ma, Pengfei Yuan, Guoheng Tang, Hongsheng Lin, Jian Dai, Wei Xue

**Affiliations:** 1 Department of Orthopedics, The First Affiliated Hospital of Jinan University, Guangzhou 510632, China.; 2 Key Laboratory of Biomaterials of Guangdong Higher Education Institutes, Guangdong Provincial Engineering and Technological Research Center for Drug Carrier Development, Department of Biomedical Engineering, Jinan University, Guangzhou 510632, China.; 3 Institute of Life and Health Engineering, Key Laboratory of Functional Protein Research of Guangdong Higher Education Institutes, Jinan University, Guangzhou 510632, China.

**Keywords:** enzyme biohybrid, light-driven pinpoint release, self-activated therapeutic cascade, tumor-enhanced penetration, erythrocyte membrane cloaking

## Abstract

Biomineralization of enzymes for *in vivo* diagnosis and treatment of diseases remain a considerable challenge, due to their severe reaction conditions and complicated physiological environment. Herein, we reported a biomimetic enzyme cascade delivery nanosystem, tumor-targeted erythrocyte membrane (EM)-cloaked iron-mineralized glucose oxidases (GOx-Fe^0^@EM-A) for enhancing anticancer efficacy by self-activated *in vivo* cascade to generate sufficient high toxic •OH at tumor site.

**Methods**: An ultra-small Fe^0^ nanoparticle (Fe^0^NP) was anchored in the inner cavity of glucose oxidase (GOx) to form iron-mineralized glucose oxidase (GOx-Fe^0^) as a potential tumor therapeutic nanocatalyst. Moreover, erythrocyte membrane cloaking delivery of GOx-Fe^0^ *in vivo* was designed to effectively accumulate ultra-small GOx-Fe^0^ at tumor site.

**Results**: GOx-Fe^0^@EM-A had satisfactory biocompatibility and light-trigged release efficiency. Erythrocyte membrane cloaking of GOx-Fe^0^@EM-A not only prolongs blood circulation but also protects *in vivo* enzyme activity of GOx-Fe^0^; Tumor targeting of GOx-Fe^0^@EM-A endowed preferential accumulation at tumor site. After NIR light irradiation at tumor site, erythrocyte membrane of GOx-Fe^0^@EM-A was ruptured to achieve light-driven release and tumor deep penetration of ultra-small nanosize GOx-Fe^0^ by the photothermal effect of ICG. Then, GOx-Fe^0^ occurred self-activated *in vivo* cascade to effectively eradicate tumor by producing the highly cumulative and deeply penetrating •OH at tumor site.

**Conclusion**: Tumor-targeted erythrocyte membrane-cloaked iron-mineralized glucose oxidase (GOx-Fe0@EM-A) exhibits a promising strategy for striking antitumor efficacy by light-driven tumor deep penetration and self-activated therapeutic cascade.

## Introduction

Inorganic-biological hybrid systems have generated considerable recent research interest, not only exploring functions and existing mechanism of living body, but also exhibiting the striking ability to remold and utilize the nature 1,2. Although living organisms can undergo biomineralization to form inorganic-biological hybrid systems in natural or unnatural environment states 3-5, few researchers have addressed the biomineralization of enzymes to extend the catalytic performance, serving massive important biological functions in organisms. Studies have shown that proteins have different binding sites on their framework and can accommodate additional metal ions 6. An ultrasmall platinum nanoparticle was anchored in glucose oxidases to enhance the enzyme activity by the photothermal effect 7. However, biomineralization of enzymes for *in vivo* treatment of diseases remains a considerable challenge, due to their severe catalytic conditions and unique three-dimensional structures.

It is well known that tumor growth and metabolism needs massive glucoses as an important energy source 8. Additionally, hydrogen peroxide (H_2_O_2_) as an endogenous reactive oxygen species (ROS) 9, 10, shows higher levels in most tumors than in normal tissues 11, exhibiting the potential as an important target for tumor therapy 12. Hence, we would modulate tumor metabolic alterations of glucoses and H_2_O_2_, harnessing glucose oxidase (GOx) to catalyze glucoses to gluconic acid and H_2_O_2_ 13-17. However, Studies have shown that in most cancer cells, the increased levels of H_2_O_2_ can promote tumor survival and proliferative pathways 18, accelerate the cancer cell mutagenesis and metastasis, and resist to cell death 19-21. Hence, H_2_O_2_ would be effectively catalyzed to generate highly reactive •OH by the ferrous ions (Fe^2+^) through Fenton reaction 22-25, which is one of highly toxic oxidants for nucleic acids peroxidation, protein degradation, and lipid damage 26-30.

Due to the relatively low potential of the Fe^3+^ / Fe^2+^ redox pair (0.77 V), the usual Fe^2+^ loaded carrier may evoke biooxidation during blood circulation and cause serious side effects on normal tissues 31. Therefore, an ultra-small Fe^0^ nanoparticle (Fe^0^NP) was anchored in the inner cavity of glucose oxidase (GOx) to form iron-mineralized glucose oxidase (GOx-Fe^0^) as a potential tumor therapeutic nanocatalyst. Firstly, glucose oxidase can catalyze glucoses into gluconic acid and H_2_O_2_ under the tumor acidic conditions. In the same time, harnessing gluconic acid to decrease pH around GOx-Fe^0^ in real time, the Fe^0^NPs can rapidly ionize into Fe^2+^ to catalyze H_2_O_2_ into highly toxic hydroxyl radical (•OH), causing irreversible oxidative damage to cellular macromolecules at near sites from side-effect damage 31, 32. Hence, the in situ generation of •OH in tumors is highly specific through self-activated *in vivo* cascade of GOx-Fe^0^.

However, how to effectively accumulate iron-mineralized glucose oxidases (GOx-Fe^0^) at tumor site remains a vital problem, due to renal clearance to ultra-small nanoparticles 33. Biomimetic NPs that combine synthetic nanoparticles with active cell membranes are getting more and more attention 34-36. Cell membranes endow nanoparticle special functions, such as decrease their clearance and long blood circulation 34, 37. In our previous study, we fabricated erythrocyte membrane-cloaked nanoparticles for chemophototherapy and gene therapy, achieving remarkable ablation of tumors and suppressed lung metastasis *in vivo* 38, 39. Hence, erythrocyte membrane (EM) would be utilized for cloaking delivery of GOx-Fe^0^ *in vivo*. More importantly, erythrocyte membrane as a natural vesicle guarantees *in vivo* circulation stability of GOx-Fe^0^ 40. Previous study showed that red blood cells as efficient microreactors could load detoxification enzymes for excellent blood detoxification *in vivo* 41. However, erythrocyte membranes lack of tumor-specific targeting capacity, which is insufficient to achieve high anticancer efficacy 42. Therefore, endowing erythrocyte membranes with excellent cancer targeting ability would show significant clinical values for enzyme delivery* in vivo*. In this study, DSPE-PEG-Angiopep-2 targeting peptide was inserted on the erythrocyte membrane. Angiopep-2 peptide is a tumor targeting moiety that specifically binds to low-density lipoprotein receptor-related proteins (LRP) receptor that are overexpressed on the surface of glioma cells 43, 44.

**Scheme 1:**
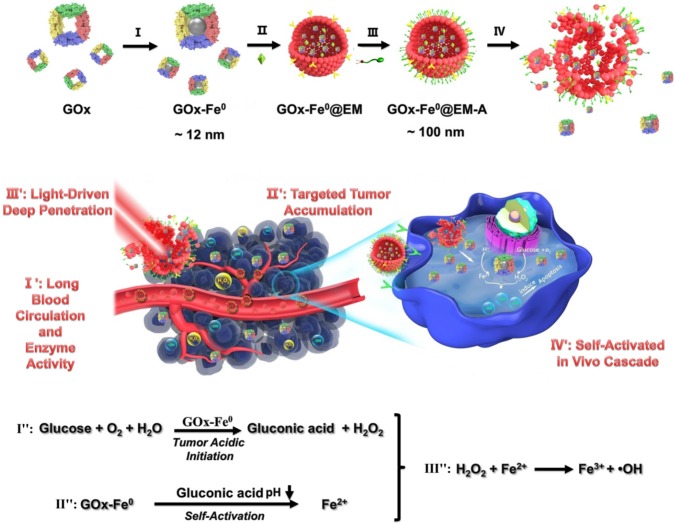
Schematic illustration for synthesis of GOx-Fe^0^@EM-A as a biomimetic enzyme cascade delivery nanosystem for I' long blood circulation and enzyme activity, II' targeted tumor accumulation, III' light-driven release and tumor deep penetration of small nanosize GOx-Fe^0^ and IV' self-activated* in vivo* cascade (I',II' and III') for chemodynamic therapy via abundant hydroxyl radicals generated at deep tumor site. I: (NH_4_)_2_Fe(SO_4_)_2_/NaBH_4_; II: Hypotonic treatment and Nano-extrusion; III: Tumor targeting designing by conjugating DSPE-PEG-Angiopep-2 into erythrocyte membrane; IV: Erythrocyte membrane rupture by photothermal effect using light irradiation.

Herein, we reported a biomimetic enzyme delivery nanosystem, tumor-targeted light-driven erythrocyte membrane-cloaked iron-mineralized glucose oxidases (GOx-Fe^0^@EM-A) by self-activated* in vivo* cascade at tumor site to generate sufficient high toxic •OH for enhancing anticancer efficacy. In order to achieve light-driven release and deep penetration of GOx-Fe^0^ at tumor site, indocyanine green (ICG) was encapsulated in erythrocyte membrane, utilizing photothermal effect to rupture the enclosed erythrocyte membrane. Due to the short half-life and lipid insolubility, the diffusion of hydroxyl radical (•OH) is limited thereby 45. Therefore, to achieve striking antitumor efficacy, highly cumulative and deeply penetrating GOx-Fe^0^ would produce •OH in the tumor deep region. At present, GOx-Fe^0^@EM-A exhibited four unique characteristics (**Scheme 1**), as summarized in the previous reports 46-48. Firstly, erythrocyte membrane cloaking maintains the nanosystem stealth and protects enzyme activity of GOx-Fe^0^. Secondly, GOx-Fe^0^@EM-A effectively accumulates at tumor site. Thirdly, light-driven release and tumor deep penetration of ultra-small nanosize GOx-Fe^0^ after NIR light irradiation. Finally, the released GOx-Fe^0^ undergoes self-activated *in vivo* cascade at tumor site.

## Results and Discussion

### Preparation and Characterization of GOx-Fe^0^@EM-A

**Figure 1:**
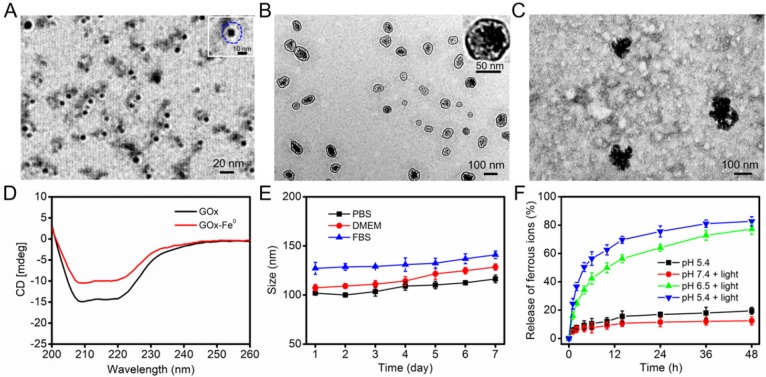
(A) TEM image of GOx-Fe^0^. A single GOx-Fe^0^ is showed in the inset, and GOx is outlined by dashed blue line. (B) TEM image of GOx-Fe^0^@EM-A. (C) TEM image of GOx-Fe^0^@EM-A after light irradiation. (D) CD spectra of GOx and GOx-Fe^0^. (E) The stability of GOx-Fe^0^@EM-A in PBS, DMEM, and FBS within 7 d. (F) The ferrous ion release from GOx-Fe^0^@EM-A at various pH with or without light irradiation. Each value represents the means ± SD (n=3).

GOx-Fe^0^ was synthesized according to the literature 7. First, GOx was complexed with ammonium iron (II) sulfate, and then, followed by in situ reduction with NaBH_4_ to prepare GOx-Fe^0^. Transmission electron microscopy (TEM) images (Figure 1A) showed that the GOx-Fe^0^ had an average size of about 12 nm. Meanwhile, the zeta potential of GOx-Fe^0^ was in accord with that of GOx, but the hydrodynamic size of GOx-Fe^0^ slightly increased, mainly due to the Fe^0^NPs successfully anchored in GOx (Figure S1a, c). Subsequently, the Fe amount of GOx-Fe^0^ was 145.8 μg/mg, determined by BCA protein assay kit and inductively coupled plasma mass spectrometry (ICP-MS). Moreover, the circular dichroism (CD) spectra result revealed that the secondary structure of GOx-Fe^0^ was almost the same as that of GOx, indicating that GOx-Fe^0^ remained the enzymatic activity of GOx (Figure 1D). Then, after GOx-Fe^0^ and ICG were passively diffused into erythrocyte membrane in a hypotonic buffer 49, 50, erythrocyte membrane was resealed with an isotonic buffer to form GOx-Fe^0^@EM by nano-extrusion. Finally, DSPE-PEG-Angiopep-2 (Figure S2) was incubated with GOx-Fe^0^@EM for 4 h to obtain Angiopep-2 conjugated biomimetic delivery system, GOx-Fe^0^@EM-A. The amount of DSPE-PEG-Angiopep-2 that was conjugated to GOx-Fe^0^@EM-A was 16.5 μg/mg. As shown in Figure 1B, GOx-Fe^0^@EM-A showed a distinct core-shell structure, demonstrating successful camouflage with erythrocyte membranes. The loading efficiency of GOx-Fe^0^ and ICG in erythrocyte membrane was 18.6% and 5.6%, respectively. The hydrodynamic sizes of GOx-Fe^0^@EM-A increased to 107 nm and the polydispersity index (PDI) of 0.27 (as shown in Figure S1a, b and Figure S3). In addition, after erythrocyte membrane cloaking, the zeta potential of GOx-Fe^0^@EM-A was -11 mV (Figure S1c), which was coincide with the zeta potential of erythrocyte membranes vesicles. These results suggested that GOx-Fe^0^ was successfully encapsulated within erythrocyte membranes. Notably, the nanosize of GOx-Fe^0^@EM-A under the physiological condition did not obviously change over 7 days (Figure 1E), which indicated the remarkable stability and potential biological applications.

Studies have shown that near-infrared light-induced local hyperthermia can trigger thermal denaturation of the integral glycoprotein on the membrane to increase the permeability of the cell membrane 51, 52. Hence, we studied the photothermal conversion capability of GOx-Fe^0^@EM-A under the 808 nm laser irradiation. The UV-vis absorption spectra showed that after five minutes laser irradiation (808 nm, 1.0 W/cm^2^), GOx-Fe^0^@EM-A exhibited similar photothermal properties to ICG (Figure S4a, b). The photothermal curve also showed that the temperature change of GOx-Fe^0^@EM-A exhibited ICG concentration dependence, and at 0.02 mg/mL ICG concentration, the solution temperature of GOx-Fe^0^@EM-A could still reached 44 °C after 5 minutes irradiation (Figure S4c). Most importantly, the TEM results exhibited obvious rupture of erythrocyte membrane under light irradiation (Figure 1C). Moreover, the size distribution changed (Figure S3d). These results suggested that near infrared light could induce erythrocyte membrane rupture to achieve light-driven release of GOx-Fe^0^. In addition, the secondary structure of GOx in GOx-Fe^0^ hardly changed under light irradiation and local heating (Figure S5). We further evaluated the release of ferrous ions from GOx-Fe^0^@EM-A under different pH and light irradiation. As shown in Figure 1F, it was observed that the ferrous ion release rate at pH 7.4 was very slow under light irradiation, and only 12.5% ion was released after 48 hours. However, in the presence of light irradiation at pH 6.5 and pH 5.4, the ferrous ion release content increased to 77.3% and 82.7%, exhibiting remarkable pH-dependent ionization. It is worth noting that the low release of ferrous ions occurred in the absence of light irradiation at pH 5.4. These results indicated that the photothermal effect could efficiently accelerate GOx-Fe^0^ release from GOx-Fe^0^@EM-A, and GOx-Fe^0^ could rapidly ionize to release ferrous ions under acidic conditions.

### Cascade Catalytic Performance of GOx-Fe^0^@EM-A

**Figure 2:**
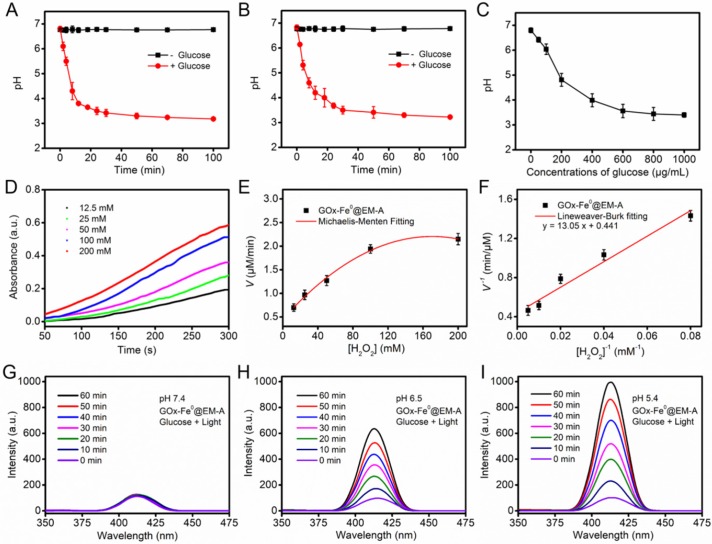
The pH value changes of GOx-Fe^0^@EM-A (A) and free GOx (B) solution in the absence and presence of glucose (1 mg/mL). (C) The pH changes from the reaction between GOx-Fe^0^@EM-A and different concentrations of glucose. (D) Time-course absorbance of GOx-Fe^0^@EM-A at varied H_2_O_2_ concentrations. (E) Michaelis-Menten kinetics and (F) Lineweaver-Burk plotting of GOx-Fe^0^@EM-A. Fluorescence spectra of OHBA induced by GOx-Fe^0^@EM-A adding glucose with light irradiation at pH 7.4 (G), 6.5 (H), or 5.4 (I).

After successfully preparing the biomimetic enzyme delivery system, GOx-Fe^0^@EM-A, its cascade catalytic performance was evaluated, compared with that of GOx-Fe^0^ and GOx. As shown in Figure 2A and B, the pH value of GOx-Fe^0^@EM-A by light irradiation and free GOx solution was constant at about pH 6.7 without addition of glucose, while after the interaction with glucose, the pH value both decreased significantly, and dropped to 3.18 and 3.22 at 100 min, respectively. In addition, due to the generated gluconic acid by enzyme catalysis, the decrease of the pH value also markedly related to glucose concentration (Figure 2C and Figure S6). It indicated that GOx-Fe^0^@EM-A still retains natural GOx activity and remains high catalytic efficiency. Moreover, when 3, 3', 5, 5'-tetramethylbenzidine (TMB) was oxidized, it showed strong absorbance at 652 nm. Hence, TMB was used to detect •OH for studying the disproportionation of H_2_O_2_ by cascade catalysis of GOx-Fe^0^ under acidic condition. As shown in Figure 2D, 100 μg/mL GOx-Fe^0^@EM-A was added to H_2_O_2_ of various concentrations and the absorbance curve was plotted to obtain an initial reaction rates. According to the Lineweaver-Burk equation (Figure 2E and F), the Michaelis-Menten constant (Km) and maximum velocity (Vmax) were calculated 23. The Km and Vmax values of GOx-Fe^0^@EM-A were 29.59 mM and 2.27 μM/min, respectively. The results indicated that the enhanced ferrous ion release of GOx-Fe^0^@EM-A under acidic environment exhibited high peroxidase-like activity and enabled to efficiently complete Fenton catalysis.

Benzoic acid (BA) could be oxidized by •OH to form strong fluorescent isomeric hydroxybenzoic acids (OHBA) 53. Therefore, we examined the generated •OH by BA fluorescence experiments to investigate the cascade catalytic performance of GOx-Fe^0^@EM-A. As shown in Figure 2G, after mixed GOx-Fe^0^@EM-A with glucose, the fluorescence spectrum of the mixture solution showed negligible change upon 808 nm light irradiation at pH 7.4. However, GOx-Fe^0^@EM-A in the presence of glucose showed significantly enhanced emission from OHBA under 808 nm light irradiation at pH 6.5 and pH 5.4 (Figure 2H and I), indicating pH-responsive generation of •OH upon enzyme cascade catalysis reaction. In contrast, the OHBA fluorescence at a lower level was observed without glucose addition or in absence of light irradiation (Figure S7). Furthermore, the OHBA fluorescence of the GOx with glucose group and ICG@EM-A with light irradiation group also exhibited negligible change (Figure S8). In addition, the oxidation of 3,3',5,5'-tetramethylbenzidine (TMB) in the presence of glucose was selected as a model catalytic reaction to investigate the enzyme cascade of GOx-Fe^0^@EM-A. As show in Figure S9, the resulting •OH will oxidize the colorless TMB to the chromogenic TMB cation radicals, showing strong absorption at 653 nm. These results confirmed that the enzyme catalysis reaction of GOx-Fe^0^@EM-A was co-initiated by glucose and acidic condition after erythrocyte membrane rupture. Moreover, it confirmed that self-activated *in vivo* cascade of GOx-Fe^0^ could be initiated in tumor microenvironment.

### *In vitro* Cascade of GOx-Fe^0^@EM-A

**Figure 3:**
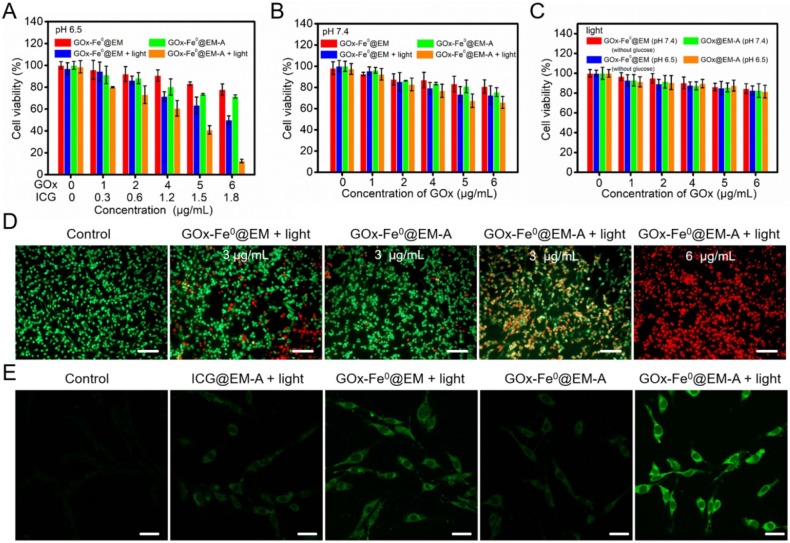
*In vitro* C6 cytotoxicity effect of GOx-Fe^0^@EM-A with or without light irradiation at pH 7.4 (A) and 6.5 (B). (C) *In vitro* C6 cytotoxicity effect of GOx-Fe^0^@EM-A (without glucose) and GOx-Fe^0^@EM-A with light irradiation at pH 7.4 and 6.5. (D) Fluorescence images of C6 cells with Calcein AM and PI staining after different treatment at pH 6.5. Scale bar: 100 µm. (E) CLSM images of ROS formation after C6 cells were incubated with different agents with or without light irradiation at pH 6.5. Scale bar: 25 µm.

Following the above results, the *in vitro* cascade of GOx-Fe^0^@EM-A on murine glioma cells (C6) was evaluated by cell cytotoxicity using a Cell Counting Kit-8 (CCK-8) assay. Upon 808 nm laser light irradiation, tumor-targeted ICG-functionalized erythrocyte membrane vesicle (ICG@EM-A) group, as a control light group for GOx-Fe^0^@EM-A, exhibited negligible cytotoxicity toward C6 cells after 24 hours of incubation at tested concentrations and different pH (Figure S10), indicating that ICG@EM-A group with low ICG concentration exhibited good cytocompatibility. Then, an acidic medium of pH 6.5 was used to mimic the tumor microenvironment, and the cytotoxicity of GOx-Fe^0^@EM-A and GOx-Fe^0^@EM was examined against C6 cells with or without 808 nm laser light irradiation. As shown in Figure 3A, at pH 6.5, there was obvious GOx-Fe^0^@EM-A's concentration-dependent cytotoxicity against C6 cells after light irradiation. For example, the viability of C6 cells treated with 6 μg/mL GOx-Fe^0^@EM-A significantly reduced to 12.5% at pH 6.5 and under light irradiation. Moreover, for 6 μg/mL GOx-Fe^0^@EM group, the cell viability was still up to 50% even at pH 6.5 and under light irradiation. It further demonstrated the specific targeting ability of Angiopep-2 conjugation. Moreover, GOx-Fe^0^@EM-A and GOx-Fe^0^@EM showed negligible effects on the viability of C6 cells under neutral conditions or without light (Figure 3A and B). Furthermore, under neutral or acidic condition, GOx-Fe^0^@EM-A (without glucose) and GOx@EM-A showed no substantial cytotoxicity against C6 cells with light irradiation (Figure 3C).

The cytotoxicity produced by GOx-Fe^0^@EM-A via enzyme cascade under acidic condition was further investigated by staining cells with calcein-AM/PI fluorescent probes to identify live and dead/late apoptotic cells. As shown in Figure 3D, most C6 cells incubated with the control group and GOx-Fe^0^@EM-A group under pH 6.5 were survived and showed strong green fluorescence. Moreover, more significant cell damage was observed after GOx-Fe^0^@EM-A with light irradiation, compared to the GOx-Fe^0^@EM with light irradiation group. As clearly seen, only minor portion of C6 cells died at GOx-Fe^0^@EM-A with light irradiation under neutral conditions (Figure S11). However, under acidic conditions, GOx-Fe^0^@EM-A induced cell apoptosis and/or necrosis under light irradiation in a dose-dependent manner, and red fluorescence increased significantly along with the concentration of GOx-Fe^0^@EM-A. These results together with the above cytotoxicity assays indicated that GOx-Fe^0^@EM-A, which specifically targets C6 cells, produces highly toxic •OH by in situ enzymatic cascade reaction under light irradiation and acidic conditions. Then, 2', 7'-dichlorofluorescein diacetate (DCFH-DA) was used as a ROS fluorescent probe to further detect •OH (Figure 3E) in C6 cells after incubation with different groups. Comparing with the blank control group, weaker green fluorescence was observed in C6 cells treated with ICG@EM-A and GOx-Fe^0^@EM under light irradiation and acidic conditions. Contrarily, cells treated with GOx-Fe^0^@EM-A with light irradiation exhibited much stronger green fluorescence than the other groups in pH 6.5 acidic conditions. This further indicated that light-driven release of GOx-Fe^0^ from the erythrocyte membrane of GOx-Fe^0^@EM-A was successfully completed and self-activated *in vitro* cascade of GOx-Fe^0^ was initiated to generate excessive •OH by Fenton reaction under acidic conditions.

### *In vitro* Tumor Penetration and *In vivo* Pharmacokinetics and Tissue Distribution of GOx-Fe0@EM-A

**Figure 4:**
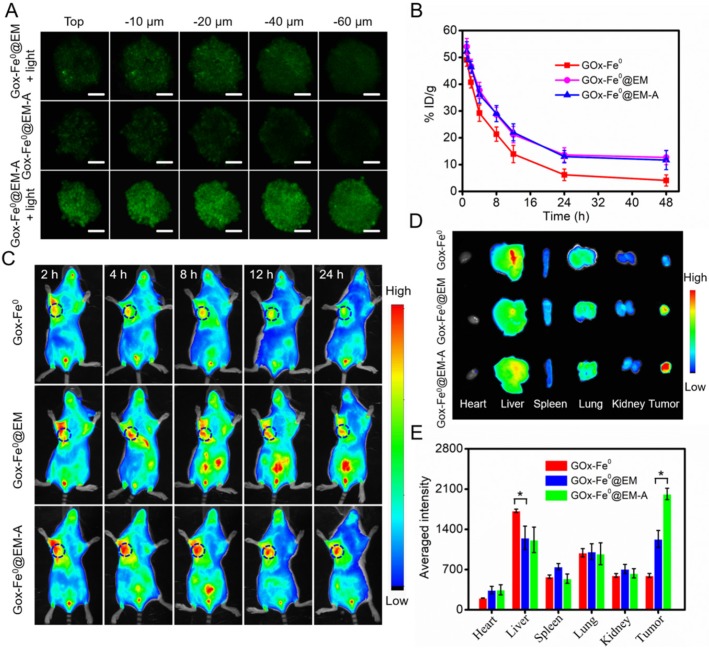
(A) 3D tumor spheroids penetration of FITC-labeled GOx-Fe^0^@EM and GOx-Fe^0^@EM-A with or without light irradiation. Scale bar was 100 µm. (B) Pharmacokinetic curves of GOx-Fe^0^, GOx-Fe^0^@EM, and GOx-Fe^0^@EM-A. (C) *In vivo* fluorescence imaging of C6 tumor-bearing mice at different times after intravenous injection of Cy5.5-labeled GOx-Fe^0^@EM and GOx-Fe^0^@EM-A, Cy5.5-labeled GOx-Fe^0^ as a control. (D) Ex vivo fluorescence imaging of major organs and tumors harvested from C6 tumor-bearing mice at 24 h post injection. (E) Semiquantitative analysis of fluorescent signals of the major organs and tumors. *P < 0.05. Each value represents means ± SD (n =3)

To evaluate the capability of tumor deep penetration of GOx-Fe^0^, C6 cells multicellular tumor sphere (MCTS) were constructed to mimic the morphology and microenvironment of solid tumor. As shown in Figure 4A, tumor spheroids were treated with FITC-labeled GOx-Fe^0^@EM-A and GOx-Fe^0^@EM for 12 hours, and ultra-small size GOx-Fe^0^ released from GOx-Fe^0^@EM-A upon light irradiation showed stronger fluorescence in the whole C6 3D tumor spheroids. However, in the absence of light irradiation, GOx-Fe^0^@EM-A (100 nm) has limited penetration ability due to larger sizes than that of GOx-Fe^0^ (12 nm). The above results indicated that light-driven released GOx-Fe^0^ from GOx-Fe^0^@EM-A exhibited enhanced penetration ability. Subsequently, we investigated the blood circulation time and tumor-targeting capability of GOx-Fe^0^@EM-A *in vivo*. First, after the intravenous (i.v.) injection of different groups, blood samples were collected at various time points, and the blood of the uninjected mice was used as a blank for ICP-AES measurement of Fe content. As shown in Figure 4B, about 11.69% ID g^-1^ and 12.67% ID g^-1^ of GOx-Fe^0^@EM-A and GOx-Fe^0^@EM remained in the blood circulation after 48 h injection, compared to GOx-Fe^0^ (4.11% ID g^-1^). GOx-Fe^0^@EM-A and GOx-Fe^0^@EM showed a significant increase in blood retention, which attributed to the camouflage of erythrocyte membrane.

Next, Cy5.5-labeled GOx-Fe^0^@EM-A was injected into C6 tumor-bearing mice through tail intravenous, and fluorescent images of mice were obtained by an *in vivo* fluorescence imaging system. As shown in Figure 4C, after i.v. injection of GOx-Fe^0^@EM-A and GOx-Fe^0^@EM, the tumor site showed apparent fluorescent signal, and the mice treated with GOx-Fe^0^@EM-A exhibited higher tumor accumulation than the GOx-Fe^0^@EM group due to the active tumor-targeting. However, due to its small nanosize, Cy5.5-labeled GOx-Fe^0^ was rapidly eliminated from the blood, and the mice tumor showed a relatively weak fluorescent signal after 24 h. The tumor-bearing mice were sacrificed after 24 h postinjection, and the main organs and tumors were collected for ex vivo fluorescence imaging. As shown in Figure 4D, Cy5.5-labeled GOx-Fe^0^ was mainly distributed in the liver and lung. Importantly, GOx-Fe^0^@EM-A group showed the strongest fluorescence at the tumor tissue compared to Cy5.5-labeled GOx-Fe^0^ and GOx-Fe^0^@EM groups. The further semiquantitative results showed that the tumor fluorescence intensity of the mice treated with GOx-Fe^0^@EM-A group was 3.38-fold and 1.64-fold than that of Cy5.5-labeled GOx-Fe^0^ and GOx-Fe^0^@EM groups, respectively (Figure 4E). Based on the above results, it suggested that the long blood circulation and remarkable tumor-specific accumulation of GOx-Fe^0^@EM-A ascribed to the camouflage and active targeting capability of Angiopep-2 functionalized erythrocyte membrane.

### Light-Driven Tumor Deep Penetration *in vivo*

**Figure 5:**
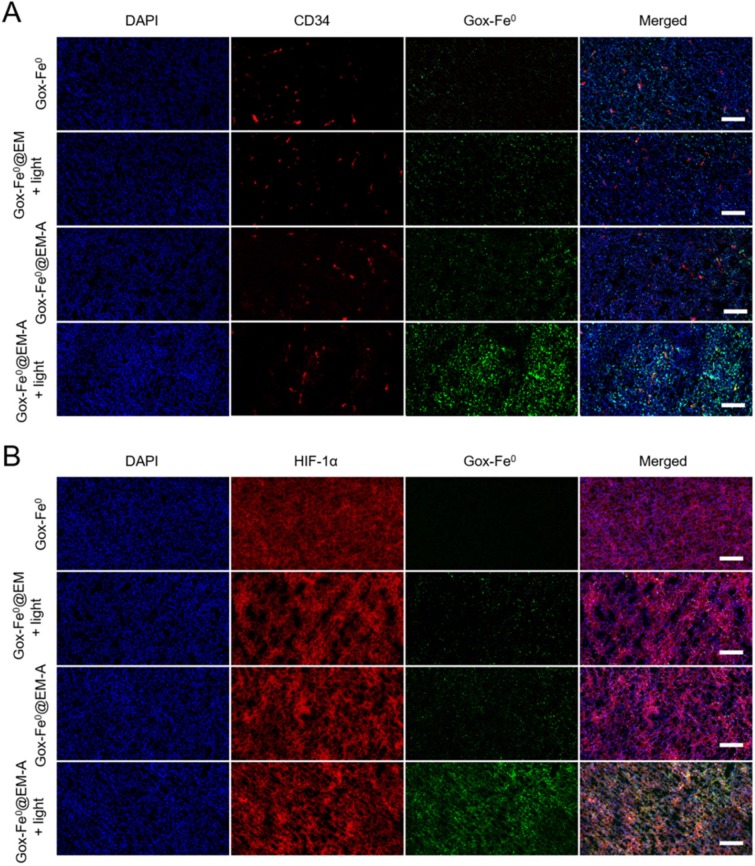
Tumor penetration of FITC-labeled GOx-Fe^0^, GOx-Fe^0^@EM and GOx-Fe^0^@EM-A with or without light irradiation into tumor vascular sparing region (A) and tumor hypoxic region (B), using confocal microscopy. Scale bar was 50 µm.

Achieving the remarkable experimental results of tumor spheroids penetration and *in vivo* imaging experiments, we subsequently studied the effect of *in vivo* tumor deep penetration of GOx-Fe^0^@EM-A driven by light irradiation in solid tumors. Tumor slices were stained with anti-CD34 and anti-HIF-1α antibody to identify tumor blood vessels and tumor hypoxia areas. As shown in Figure 5, Deep penetration of FITC-labeled GOx-Fe^0^, GOx-Fe^0^@EM and GOx-Fe^0^@EM-A with or without light irradiation into tumor vascular sparing region (A) and tumor hypoxic region (B) was evaluated by the immunofluorescence staining study. GOx-Fe^0^ or GOx-Fe^0^@EM-A groups showed a weak fluorescent signal at tumor blood vessel site. The weak signal of GOx-Fe^0^ was due to the low tumor accumulation from tumor blood vessels, whereas the GOx-Fe^0^@EM-A could not easily penetrate into the deep tumor space, due to the large nanosize (~100 nm). However, after membrane rupture driven by light irradiation, GOx-Fe^0^@EM-A and GOx-Fe^0^@EM released small nanosize GOx-Fe^0^ (~12 nm) previously encapsulated. Meantime, both of the two groups showed a stronger fluorescence signal at tumor site than that without light irradiation, and the GOx-Fe^0^@EM-A group showed higher tumor accumulation than the GOx-Fe^0^@EM group, exhibiting uniform perfusion in the tumor stroma. In addition, after light irradiation, the fluorescence intensity of GOx-Fe^0^ released from GOx-Fe^0^@EM-A significantly increased at a greater distance from the CD34-labeled tumor blood vessels (Figure 5A) and in the HIF-1α-labeled tumor hypoxia region (Figure 5B). These results indicated that the tumor-targeting and the light-driven membrane rupture of GOx-Fe^0^@EM-A enhanced tumor accumulation and deep penetration of GOx-Fe^0^ released, due to small nanosize effect at tumor blood vessel site.

### *In vivo* Cascade of GOx-Fe^0^@EM-A for Tumor Treatment

**Figure 6:**
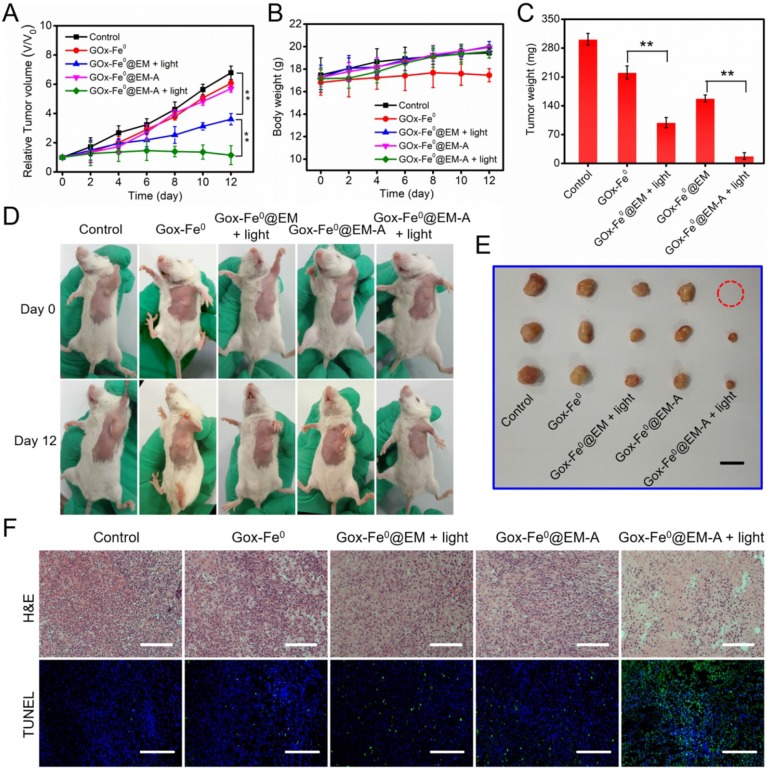
(A) Relative tumor volume and (B) body weight changes in 12 days after various treatments. **P < 0.01. (C) The average tumor weight after various treatments.**P < 0.01. (D) Photographs of C6 tumor-bearing BALB/c mice on Day 0 and Day 12. (E) Representative photographs of tumors. (F) H&E and TUNEL stained tumor slices after different treatments. The H&E scale bars represent 100 µm. The TUNEL scale bars represent 200 µm.

Following the catalytic efficacy* in vitro* and tumor targeted accumulation and deep penetration, the antitumor efficacy *in vivo* of GOx-Fe^0^@EM-A was evaluated in two C6 tumor-bearing mice model with different immune capability. When the tumor volume reached 80 mm^3^, the tumor mice were randomly divided into 5 groups, and different formulations and treatments were administered through tail i.v. injection. As shown in Figure S12, tumors injected with PBS and irradiated, the tumor temperature only increases to 36 °C, which was unable to cause tumor ablation. However, after laser irradiation with 5 minutes, tumor temperature was increased to 43.3 °C were observed for tumors injected with GOx-Fe^0^@EM-A, which effectively increased cell membrane permeability and promoted the release of GOx-Fe^0^. The tumor growth curve in tumor-bearing BALB/c mice model indicated that the PBS group showed a rapid tumor growth trend during treatment (Figure 6A). As expected, tumor growth hardly inhibit after injection of GOx-Fe^0^ and GOx-Fe^0^@EM, probably attributing to limited tumor accumulation by GOx-Fe^0^ and GOx-Fe^0^@EM. In contrast, the GOx-Fe^0^@EM with light irradiation group exhibited the effective therapeutic efficacy. More importantly, GOx-Fe^0^@EM-A with light irradiation group induced the remarkable tumor growth inhibition within 12 days, attributing to the preferential tumor accumulation and in situ •OH production ability of GOx-Fe^0^@EM-A. Figure 6D showed representative photographs of mice after different treatments. Tumor-bearing mice were sacrificed at the end of treatment, and tumor tissues collected and weighed (Figure 6C and E). These results showed that the GOx-Fe^0^@EM-A with light irradiation group achieved almost complete tumor eradication. During the treatment period, except for the slow growth of the mice weight of GOx-Fe^0^ groups, no significant discrepancy in the body weight of the other groups was observed (Figure 6B), indicating that erythrocyte membrane-cloaked enzyme delivery nanosystems hardly influenced the health of the mice. Next, hematoxylin and eosin (H&E) and terminal deoxynucleotidyl transferase-mediated dUTP-biotin nick end labeling (TUNEL) stained tumor slices were used to further examine the therapeutic efficacy* in vivo* of GOx-Fe^0^@EM-A. As shown in Figure 6F, severe damage and aggravation of tumor cells was observed in the GOx-Fe^0^@EM-A with light irradiation group. In contrary, other groups induced negligible cell apoptosis.

**Figure 7:**
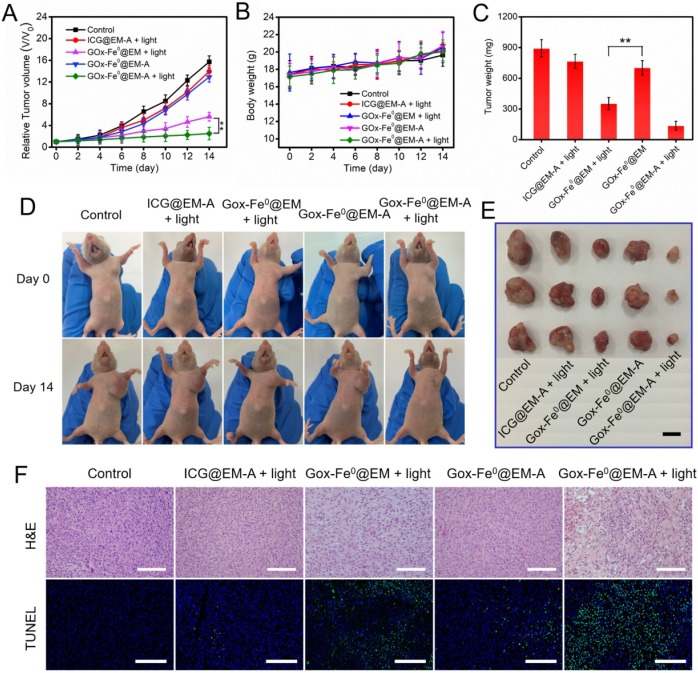
(A) Relative tumor volume and (B) body weight changes in 12 days after various treatments. **P < 0.01. (C) The average tumor weight after various treatments.**P < 0.01. (D) Photographs of C6 tumor-bearing BALB/c nude mice on Day 0 and Day 14. (E) Representative photographs of tumors. (F) H&E and TUNEL stained tumor slices after different treatments. The H&E scale bars represent 100 µm. The TUNEL scale bars represent 200 µm.

To further confirm the above therapeutic results, we constructed another C6 tumor xenograft for* in vivo* therapeutic experiments (Figure 7). The antitumor studies in C6 tumor-bearing BALB/c nude mice model revealed that GOx-Fe^0^@EM-A with light irradiation group showed similar striking tumor inhibition, compared to that in BALB/c mice model. No significant changes were observed in mouse body weight during the treatment period. The substantial therapeutic efficacy *in vivo* of GOx-Fe^0^@EM-A with light irradiation was further proved. In addition, H&E staining of all groups against the major organs (heart, liver, spleen, lung, and kidney) showed no obvious organ damage and inflammatory changes compared to the PBS group (Figure S13A). Blood biochemical analysis showed (Figure S13B) that the GOx-Fe^0^ group showed reduced plasma glucose (GLU) compared to the PBS group, probably due to free alive GOx-Fe^0^ promoting catalytic decomposition of glucose in the blood. However, the GOx-Fe^0^@EM-A and GOx-Fe^0^@EM group had no noticeable changes in biochemical parameters of aspartate aminotransferase (AST), albumin globulin (ALB), urea nitrogen (BUN), uric acid (UA), and GLU, demonstrating no damage to liver, kidney and blood glucose function. These results indicate that tumor-targeted GOx-Fe^0^@EM-A exhibited sufficient tumor accumulation, and then through light-driven enzyme biohybrid' release and tumor acidic condition to complete cascade sequential catalysis to generate large amount of highly toxic •OH and achieve efficient tumor growth inhibition, showing minimal toxicity to normal major organs at the test dose.

## Conclusion

In summary, we developed a biomimetic enzyme cascade delivery nanosystem, tumor-targeted light-driven erythrocyte membrane-cloaked iron-mineralized glucose oxidases (GOx-Fe^0^@EM-A) by self-activated *in vivo* cascade of GOx-Fe^0^ at tumor deep site to generate sufficient high toxic •OH for enhancing anticancer efficacy. Due to the biomimetic surface functionalization of erythrocyte membrane, GOx-Fe^0^@EM-A showed prolonged blood circulation and enhanced tumor accumulation. Moreover, at tumor site light-driven erythrocyte memebrane rupture by photothermal effect of ICG enhanced the pinpoint release and tumor tissue penetration of small nanosize GOx-Fe^0^. More importantly, the released GOx-Fe^0^ undergoes self-activated *in vivo* cascade to effectively produce the highly cumulative and deeply penetrating •OH to effectively eradicate tumor. *In vitro* and *in vivo* studies indicated that GOx-Fe^0^@EM-A exhibited dual-enzyme-like activity of glucose oxidase and peroxidase, and showed highly effective anticancer inhibition by enzyme cascade catalysis at the tumor deep site, with negligible side effects. In short, our rationally designed biomimetic enzyme cascade delivery nanosystem exhibits a new strategy of chemodynamic therapy for effective tumor eradication and provides valuable insights in nanomedicine to develop safe and highly efficient biomimetic nanocatalytic systems for various malignant tumor treatments.

## Experimental Section

### Characterization

Transmission electron microscope (TEM) images were obtained with Hitachi H-7650 operated under 80 kV. Dynamic light scattering (DLS) and zeta potential were conducted on a Zetasizer Nano ZS particle analyzer (Malvern Instruments Limited). UV-Vis absorption spectra were performed on a Shimadzu UV-2550 UV-vis spectrophotometer. The fluorescence spectra were measured using a 970CRT spectrophotometer. The CD spectra were carried out using a chirascan-plus spectropolarimeter (Applied Photophysics, UK).

### Acidic-dependent ferrous ions release from GOx-Fe^0^@EM-A

1 mL of GOx-Fe^0^@EM-A solution (with Fe concentration at 1 mg mL^-1^) was sealed into a dialysis bag (molecular weight cutoff = 3000 Da) against 10 ml of the PBS buffer solution (pH 7.4, pH 6.5 and pH 5.4) with or without 808 nm laser (1 W/cm^2^) irradiation, and gently shaken at 37 °C with shaker at 100 rpm. At predetermined time intervals, 0.2 mL of solution was withdrawn and displaced with fresh solution. The released Fe concentration was analyzed by ICP-MS.

### Michaelis-Menten kinetics

For a typical peroxidation reaction, various concentrations of H_2_O_2_ (12.5 mM, 25 mM, 50 mM 100 mM, and 200 mM) was mixed with TMB (1 mM) and GOx-Fe^0^@EM-A (100 μg/mL) in NaAc buffer solution (20 mM pH = 5.2). The chromogenic reaction (λ = 650 nm) was monitored with UV-vis spectrometer. The Michaelis-Menten kinetic curve can then be obtained by converting the absorbance changes to the initial velocity through Beer-Lambert law and plotting against the H_2_O_2_ concentration. The Michaelis-Menten constant (KM) and the maximum velocity Vmax were calculated by a linear double reciprocal plot (Lineweaver-Burk fitting).

### Hydroxyl radical (•OH) generated assessment of GOx-Fe^0^@EM-A

Benzoic acid (BA) was dissolved in PBS at different pH (7.4, 6.5, and 5.4). In a typical process, GOx-Fe^0^@EM-A (0.3 mL, 100 μg/mL) was added into a mixture solution (3 mL) containing glucose (2 mM) and different pH of BA (2 mM). After 808 nm (1 W/cm^2^) light irradiation, the fluorescence spectrum of BA was monitored for 60 minutes and the emission intensity of OHBA at 410 nm was plotted against time.

### Cell culture

C6 cancer cells were obtained from American Type Culture Collection (ATCC) and cultured in Dulbecco modified eagle medium (DMEM) containing 4500 mg/L glucose, 10% fetal bovine serum and 1% penicillin/streptomycin. The cells were maintained at 37 °C in humidified atmosphere with 5% CO_2_.

### Cytotoxicity Measurements

C6 cells were seeded in a 96-well plate with a cell density of 5000 cells per well for 12 h. Then cell culture medium were replaced with different products under different GOx or ICG concentrations in DMEM high-glucose medium (pH was adjusted to 7.4 and 6.5 by the addition of HCl) for 24 h. After 4 h incubation, cells were illuminated by 808 nm light irradiation (1 W/cm^2^) for 5 min. After another 20 h incubation, the cells were mixed with 10% CCK-8 containing DMEM high-glucose medium. CCK-8 could measure by a microplate reader (MultiskanMk3, USA) at 450 nm.

In order to further studied the viable cells and dead cells after cytotoxicity measurements, the cells were incubated with GOx-Fe^0^@EM (3 μg/mL of GOx), GOx-Fe^0^@EM-A (3 μg/mL of GOx), or GOx-Fe^0^@EM-A (6 μg/mL of GOx), and then with or without irradiated by 808 nm laser (1 W/cm^2^). The cell culture medium was removed. After staining with Calcein AM/PI for 20 min, the cells were washed and monitored by inverted fluorescent microscope (Axio Observer A1, Zeiss, Germany).

### Detection of Intracellular ROS Production

C6 cells were seeded in 2 cm confocal microscopy dish (1×10^5^ cells/well) for 12 h. Subsequently, the cells were incubated with RI (4 μg/mL of ICG), GOx-Fe^0^@EM (15 μg/mL of GOx), or GOx-Fe^0^@EM-A (15 μg/mL of GOx), and then with or without irradiated by 808 nm laser (1 W/cm^2^). After that, the cells were washed with PBS and treated with DCFH-DA (10 μM) for 30 min. Finally, the cells were detected with confocal fluorescence microscope by an excitation of 488 nm.

### Penetration of GOx-Fe^0^@EM-A (with or without light) in C6 Spheroid

C6 cells were seeded on an ultralow adsorption 6-well plate at a density of 2 × 10^5^ cells per dish. The culture medium was replaced every other day. C6 MCTs formed spontaneously after 5 days. Then the MCTS were treated with the FITC-loaded GOx-Fe^0^@EM and GOx-Fe^0^@EM-A nanoparticles, followed by light irradiation with or without 808 nm (1 W/cm^2^) for 5 min. After another 8 h incubation, the spheroids were washed three times and observed with CLSM from the top to the middle of spheroid.

### *In vivo* Pharmacokinetics

Female BALB/c mice bearing C6 tumor xenografts (n=3) received intravenous injected with of GOx-Fe^0^, GOx-Fe^0^@EM, or GOx-Fe^0^@EM-A at a dose of 2 mg Fe kg^-1^. At different time points after the injection, venous blood was collected from the tail veins. The Fe ions contents in blood were determined by ICP-MS.

### *In vivo* Fluorescence Imaging

The female Balb/c mice with C6 tumors were performed *in vivo* imaging studies. The mice were intravenously injected with Cy5.5-labeled GOx-Fe^0^@EM or GOx-Fe^0^@EM-A (with equivalent Cy5.5 of 0.4 μmol kg^-1^), and then the fluorescent images were observed using VIS-FL *in vivo* imaging system (Cambridge Research & Instrumentation; Woburn, MA) at different time intervals. After the 24 h post-injection, the mice were sacrificed and the tumors as well as normal tissues were collected for ex vivo imaging. The region-of-interests were analyzed by using Living Image software.

## Supplementary Material

Supplementary experimental section and figures.
