# Supplementary material for: Self-activated in vivo therapeutic cascade of erythrocyte membrane-cloaked iron-mineralized enzymes
Source: Theranostics. 2020 Jan 12;10(5):2201–14. doi: 10.7150/thno.39621 (PMC7019169; doi:10.7150/thno.39621)
Supplement: Supplementary file 1 — Supplementary experimental section and figures. [file thnov10p2201s1.pdf]

Supporting Information

Self-Activated *in Vivo* Therapeutic Cascade of Erythrocyte Membrane-Cloaked Iron-Mineralized Enzymes

Wen Liu[1,2], *Miao-Liang Ruan*[2], *Lamei Liu*[2], *Xin Ji*[2], *Yandong Ma*[2], *Pengfei Yuan*[2], *Guoheng Tang*[2], *Hongsheng Lin* [1,*], *Jian Dai*[1,2*], *Wei Xue*[1,2,3,*]

Experimental Section

Materials. Glucose oxidase (GOx) was purchased from *Bioroyee Co. Ltd.,* Beijing, China. Ammonium iron (II) sulfate and sodium borohydride ($NaBH_4$) were obtained from Sigma-Aldrich Corp. Indocyanine green (ICG) was obtained from Tokyo Chemical Industry Co., Ltd. 1,2-distearoyl-sn-glycero-3-phosphoethanolamine-N-[maleimide(polyethylene glycol)] (DSPE-PEG2000-MAL) was purchased from Jinpan Biotech Co. Ltd., China. Angiopep-2 was purchased from Yaoqiang Biotechnology Co., Ltd., Shanghai, China. glucose was purchased from Guangzhou Chemical Reagent Factory. Benzoic acid (BA) and Tris(2-carboxyethyl)phosphine hydrochloride (TCEP) was purchased from Aladdin Industrial Co. Ltd., Shanghai, China. The BCA protein assay kit was obtained from Invitrogen Corp. 2′,7′-dichlorofluorescein diacetate (DCFH-DA) was obtained from Sigma-Aldrich Corp. The CCK-8 was purchased from *Dojindo Corp.*, Kumamoto, Japan. Calcein-AM and propidium iodide (PI) were purchased from Beyotime Inst. Biotech.

Synthesis of GOx-Fe^0. Ammonium iron(II) sulfate was used as a source of iron nanoparticles in the reaction system. First, 0.5 mg GOx and 1 mL ammonium iron (II) sulfate (2.5 mM) were dissolved in 10 mL Milli-Q water, and the solution was magnetically stirred under N_2 protection for 0.5 h. Then, 2.5 mL $NaBH_4$ (7.5 mM)

was dropwise added to the solution at a constant rate of 0.1 ml min^{-1}. 2 h later, the products were purified by a centrifugal filter device (30 kDa MWCO) (Millipore) for three times.

Synthesis of Angiopep-2-PEG-DSPE. 20 mg DSPE-PEG-MAL was dispersed in 2% EDTA phosphate buffer (3 mL), and then the mixed 20 mg Angiopep-2 and 0.1 mL TCEP (1 mg/mL) in 2% EDTA phosphate buffer (3 mL) were added to the solution with stirring for 4 h. Afterward, dialysis was performed to remove the excess materials.

Synthesis of erythrocyte membrane. The fresh blood was collected from female BALB/c mice. The whole blood was centrifuged at 2500 rpm for 5 min at 4 ℃ to remove the plasma and the buffy coat. The resulting erythrocyte membranes were washed with cold PBS three times and then dissolved in cold hypotonic buffer for hemolysis at 4 ℃. After 4 h, the supernatant hemoglobin was removed by centrifugation at 3500 r/min for 5 minutes at 4 ℃. The collected erythrocyte ghosts were washed three times with cold PBS buffer for purification. Then, the final erythrocyte membranes were re-dispersed in saline at -80 ℃ for further use.

Synthesis of GOx-Fe^0@EM. A certain amount of GOx-Fe^0 (3 mg/mL) and ICG (1 mg/mL) were added into erythrocyte membranes (10 mg/mL) solution, and the mixture was magnetically stirred at 4 ℃ overnight, and then the supernatant was removed by centrifugation at 3500 rpm for 5 min to remove the excess GOx-Fe^0 and ICG. The collected GOx-Fe^0@EM was washed three times with cold PBS buffer. Afterward, the GOx-Fe^0@EM was extruded through polycarbonate porous

membranes (100 nm) for at least 11 passes using an Avanti mini extruder (AvantiPolar Lipids).

Synthesis of GOx-Fe^0@EM-A. A certain amount of GOx-Fe^0 (3 mg/mL) and ICG (1 mg/mL) were added into erythrocyte membranes (10 mg/mL) solution, and the mixture was magnetically stirred at 4 ℃ overnight, and then the supernatant was removed by centrifugation at 3500 rpm for 5 min to remove the excess GOx-Fe^0 and ICG. The collected GOx-Fe^0@EM was washed three times with cold PBS buffer. Then, Angiopep-2-PEG-DSPE was added into GOx-Fe^0@EM solution and reacted for 4 h at 4 ℃. The supernatant was removed by centrifugation at 3500 rpm for 5 min to remove the excess Angiopep-2-PEG-DSPE. The collected GOx-Fe^0@EM-A was washed three times with cold PBS buffer. Afterward, the GOx-Fe^0@EM-A was extruded through polycarbonate porous membranes (100 nm) for at least 11 passes using an Avanti mini extruder (AvantiPolar Lipids).

In Vivo Tumor Penetration of GOx-Fe^0@EM-A (with or without light irradiation). The female Balb/c mice with C6 tumors were performed in vivo tumor penetration studies. The Fitc-labeled GOx-Fe^0, GOx-Fe^0@EM and GOx-Fe^0@EM-A were prepared and injected into the C6 tumor xenografts via the tail vein in vivo. After 6 h, the mice were irradiated by 808 nm laser (1 W/cm^2) for 5 min or without any treatment, and the tumor was collected 4 h later. The tumor sections were stained with anti-CD34 and anti-HIF-1α antibody for CLSM examination.

In Vivo Therapeutic Efficacy. To establish the tumor xenograft model, the female BALB/c mice and Male BALB/c nude mice were subcutaneously inoculated with 3 $\times 10^6$ C6 cells suspended in 100 μL of PBS. To evaluate antitumor effects, C6 tumor-bearing female BALB/c mice or Male BALB/c nude mice were randomly divided into five groups (n = 5 mice/group): i) control, ii) GOx-Fe^0, iii)

$GOx-Fe^0$@EM + light, iv) $GOx-Fe^0$@EM-A, v) $GOx-Fe^0$@EM-A + light. When the tumor volume reached about 80 mm^3, the mice were intravenously injected with Saline (200 μL), $GOx-Fe^0$ (200 μL, 3 mg/kg GOx), $GOx-Fe^0$@EM (200 μL, 3 mg/kg GOx), or $GOx-Fe^0$@EM-A (200 μL, 3 mg/kg GOx). After 6 h, tumors were irradiated by 808 nm laser (1 W/cm^2) for 5 min or without any treatment. The treatment was repeated every three days for three times. The tumor volume and body weight were measured every other day. Tumor volume (V) was calculated as follows: $V = W^2 \times L/2$, where W and L represented the tumor length and tumor width, respectively. At day 12, the mice were sacrificed, the tumors and the main organs were collected, and fixed using 4% formalin, embedded in paraffin, sliced for H&E and TUNEL apoptosis staining and observed using an optical microscope. The blood of mice was taken out for evaluating the level of GLU, AST, ALB, BUN, and UA.

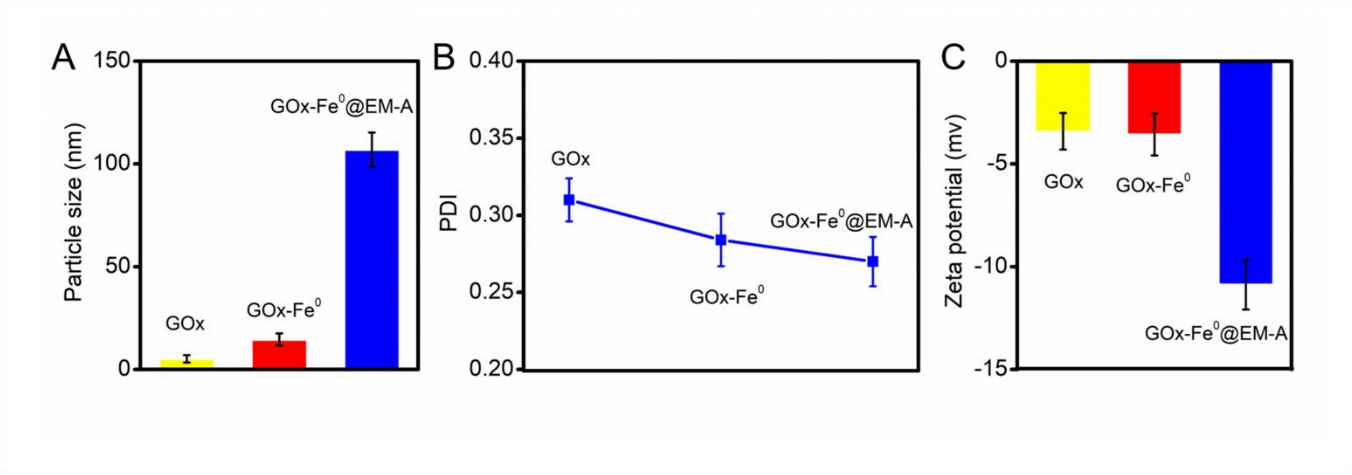

Figure S1. Particle size (A), PDI (B), and ζ-potential (C) of GOx, GOx-Fe^0, and GOx-Fe^0@EM-A.

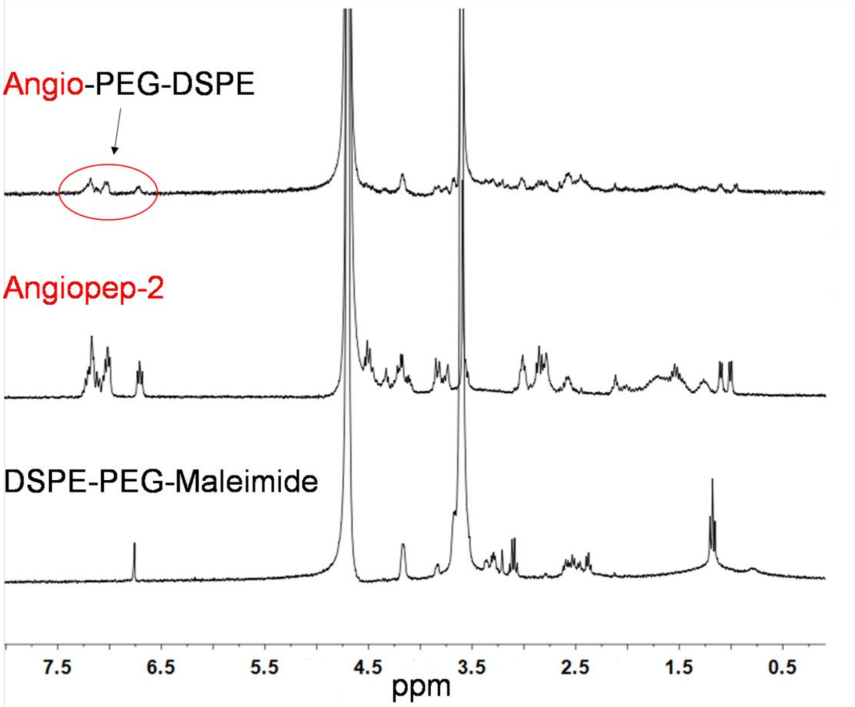

Figure S2.^{1}H-NMR spectrum of Angio-PEG-DSPE measured in D_2O at 25 ℃.

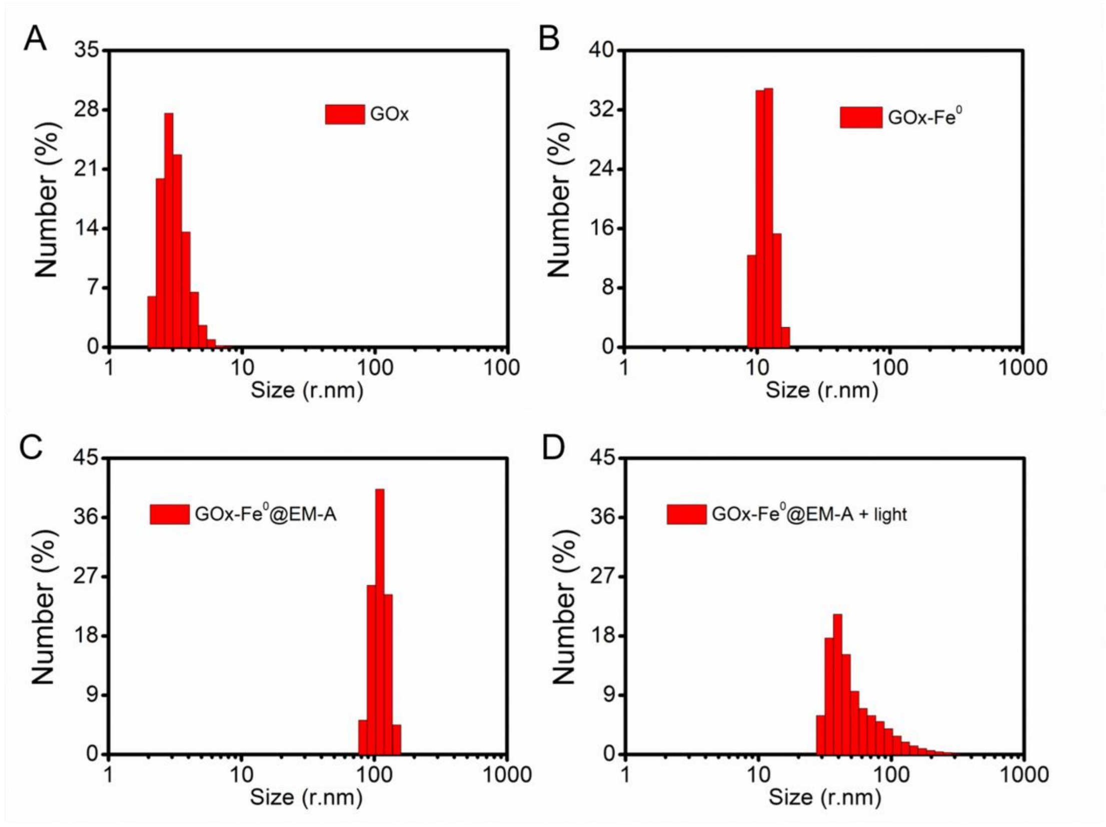

Figure S3. Hydrodynamic size distribution of GOx (A), GOx-Fe^{0} (B), GOx-Fe^{0}@EM-A (C), and GOx-Fe^{0}@EM-A after 808 nm light irradiation (D).

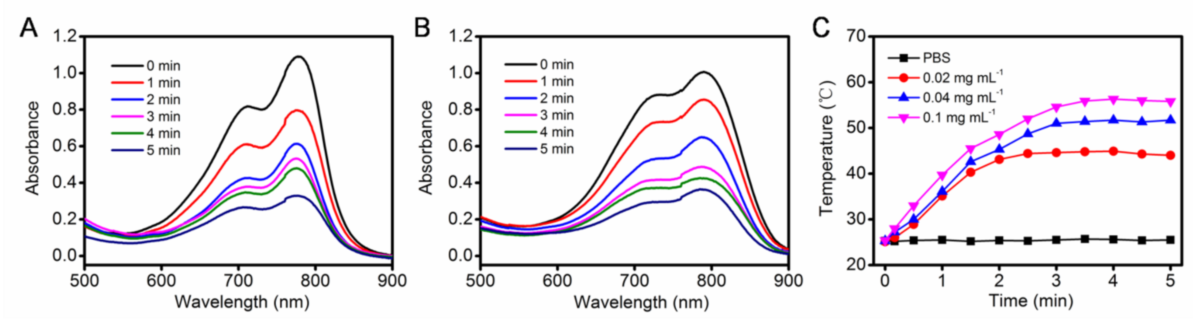

Figure S4. UV-Vis spectra of ICG (A) and GOx-Fe^{0}@EM-A (B) after 808 nm light irradiation. (C) The temperature changes of GOx-Fe^{0}@EM-A at different concentrations under light irradiation.

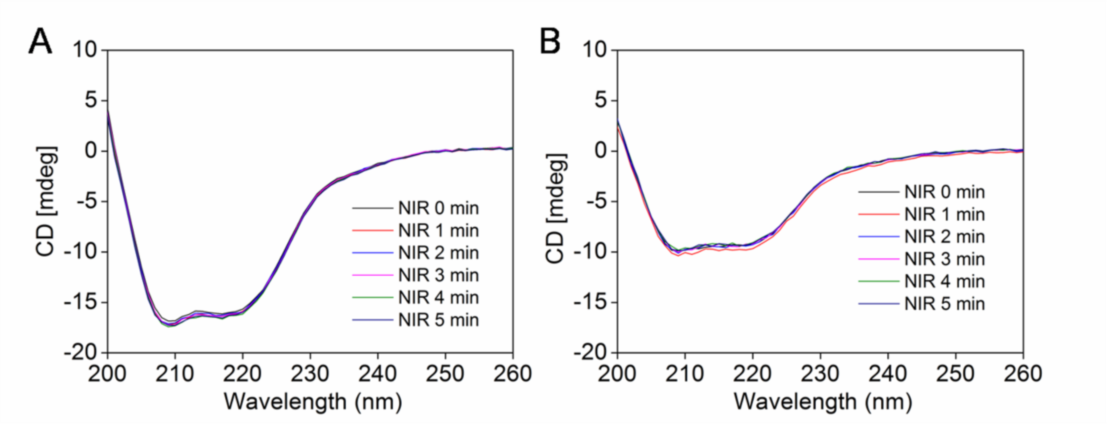

Figure S5. CD spectra of GOx (A) and GOx-Fe^0 (B) recorded after light irradiations.

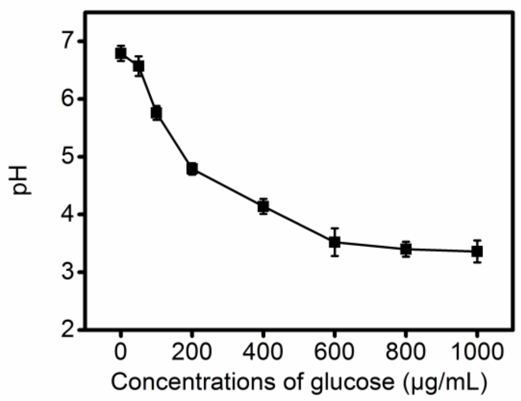

Figure S6. The pH changes from the reaction between GOx and different concentrations of glucose.

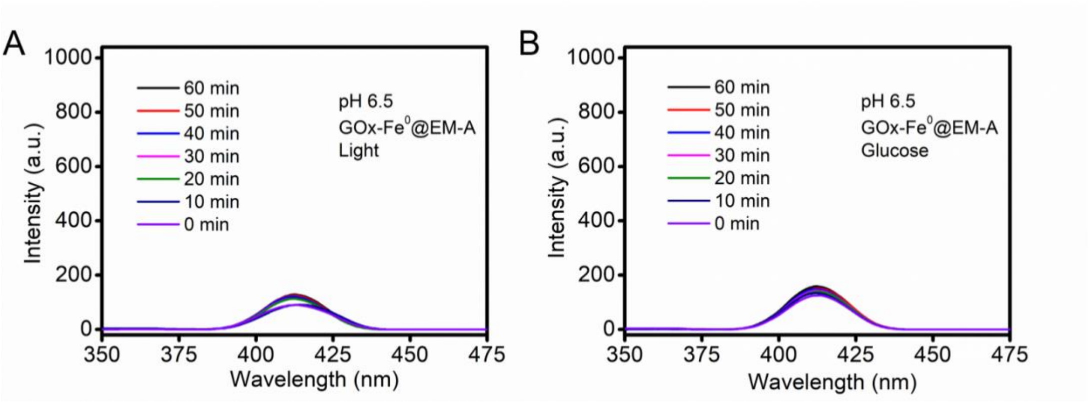

Figure S7. (A) Fluorescence spectra of OHBA induced by GOx-Fe^{0}@EM-A under light irradiation at pH 6.5. (B) Fluorescence spectra of OHBA induced by GOx-Fe^{0}@EM-A and glucose at pH 6.5.

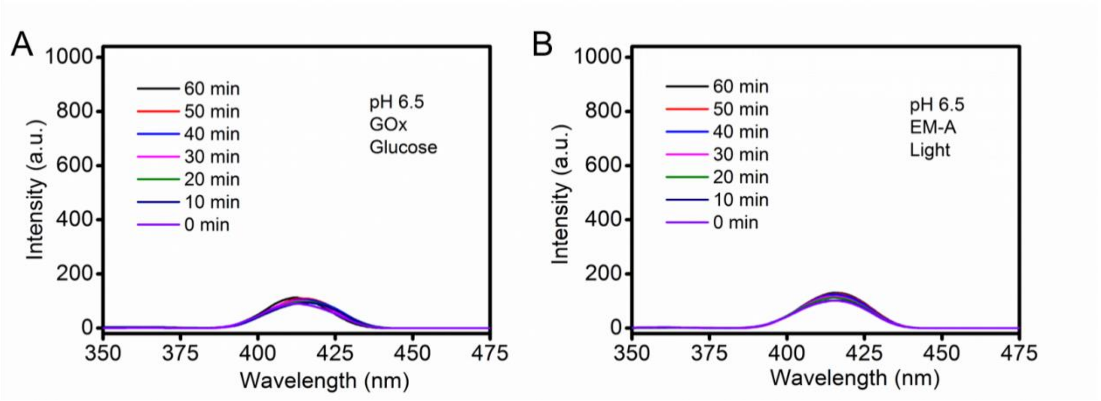

Figure S8. (A) Fluorescence spectra of OHBA induced by GOx and glucose at pH 6.5. (B) Fluorescence spectra of OHBA induced by EM-A under light irradiation at pH 6.5.

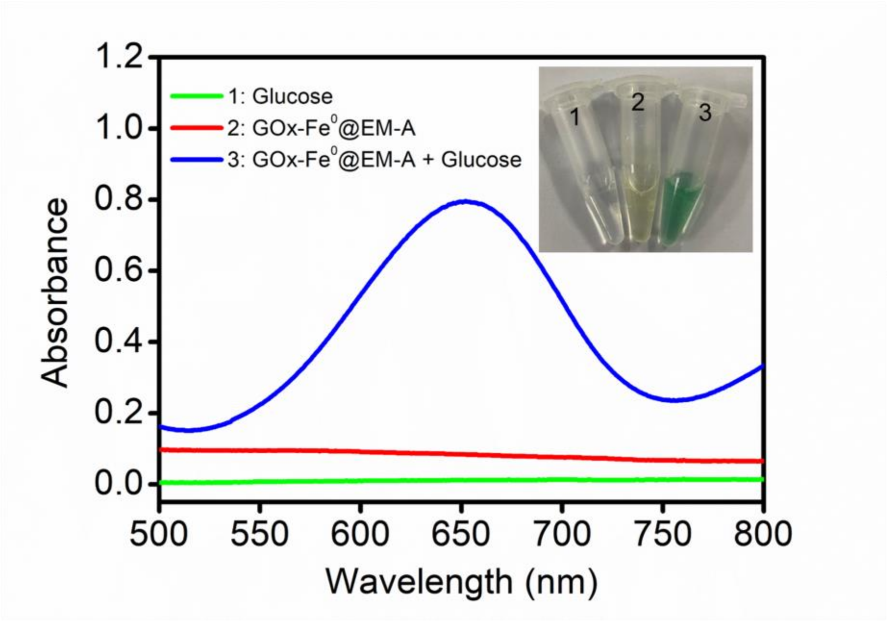

Figure S9. UV/Vis absorbance spectra and visual color changes for three different samples from TMB-specific assay.

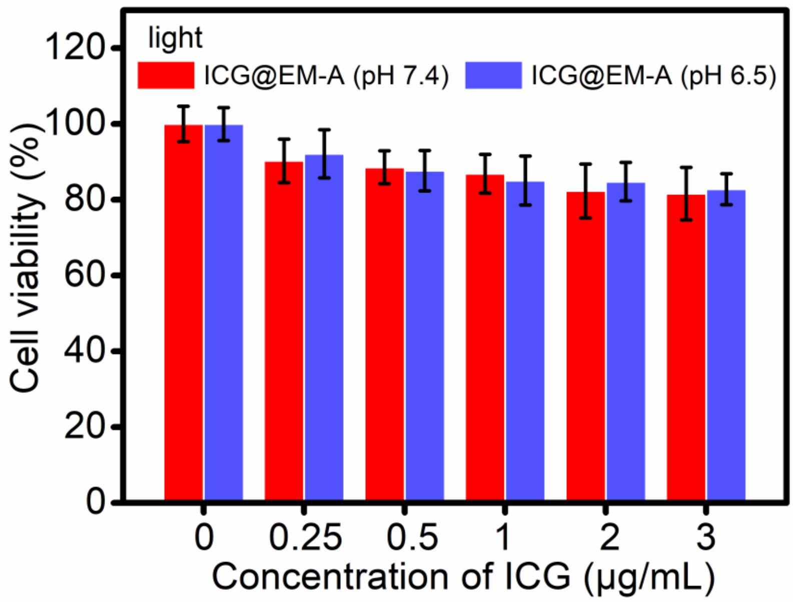

Figure S10. In vitro C6 cytotoxicity effects of ICG@EM-A with light irradiation at pH 7.4 and 6.5.

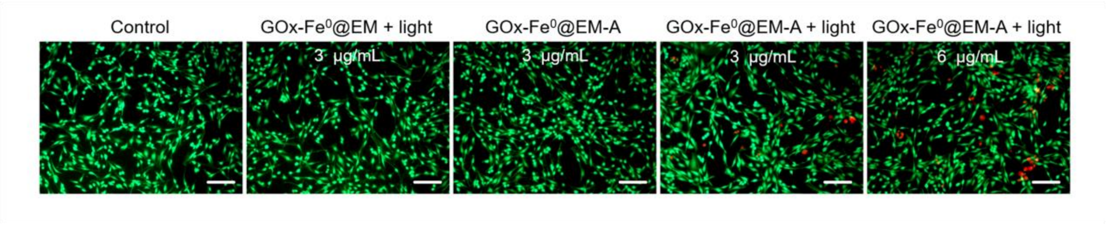

Figure S11. Fluorescence images of C6 cells with Calcein AM and PI staining after different treatment at pH 7.4. Scale bar: 100 μm.

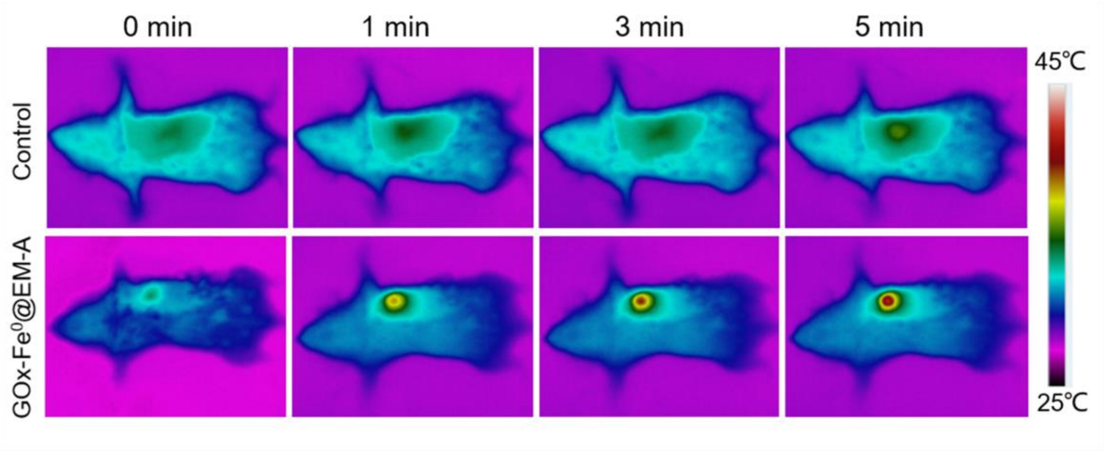

Figure S12. The IR images of C6 tumor-bear mice with injected of PBS, GOx-Fe^0@EM-A under NIR laser irradiation.

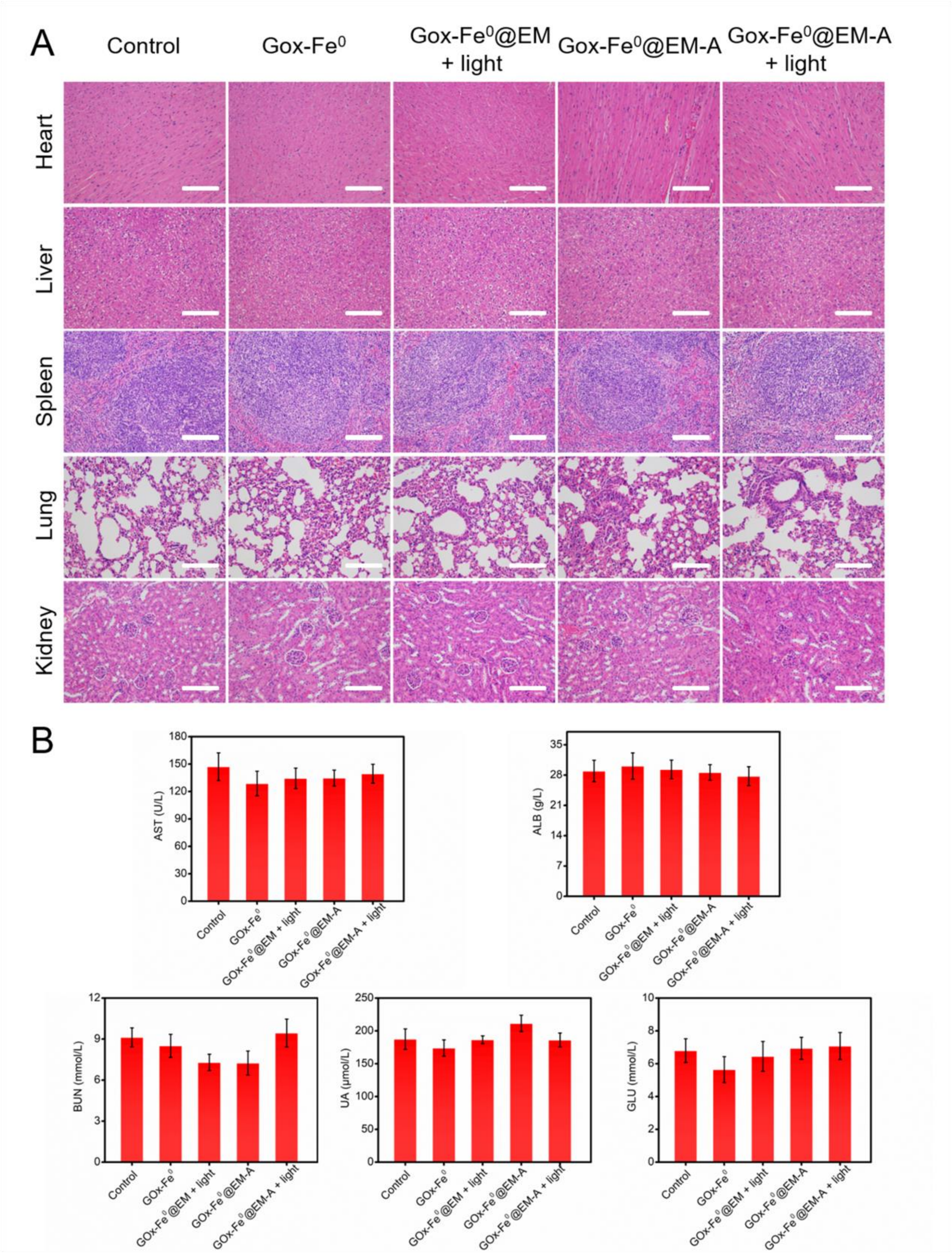

Figure S13. *In vivo* systemic toxicity evaluation. (A) H&E staining of major organs after treatment with different samples (Scale bar = 100 μm). (B) Blood biochemistry analysis of various groups after 12 days treatment: AST, ALB, BUN, UA, and GLU.
